# Isotopic compositions of ^236^U, ^239^Pu, and ^240^Pu in soil contaminated by the Fukushima Daiichi Nuclear Power Plant accident

**DOI:** 10.1038/s41598-017-13998-6

**Published:** 2017-10-19

**Authors:** Guosheng Yang, Hirofumi Tazoe, Kazuhiko Hayano, Kumiko Okayama, Masatoshi Yamada

**Affiliations:** 1 Department of Radiation Chemistry, Institute of Radiation Emergency Medicine, Hirosaki University, 66-1 Hon-cho, Hirosaki, Aomori, 036-8564 Japan; 2 Division of Nuclear Technology and Applications, Institute of High Energy Physics, Chinese Academy of Sciences; Beijing Engineering Research Center of Radiographic Techniques and Equipment, Beijing, 100049 China; 3 Mutsu Analytical Sciences Laboratory, Japan Chemical Analysis Center, 4-24 Minatomachi, Mutsu, Aomori, 035-0064 Japan

## Abstract

Six years after the Fukushima Daiichi Nuclear Power Plant (FDNPP) accident, data for ^236^U and ^236^U/^238^U have remained limited to a few heavily contaminated samples. In the present study, activities of ^236^U, ^239^Pu, and ^240^Pu, along with other U isotopes in 46 soil samples both heavily and lightly contaminated by this accident were measured by inductively coupled plasma–mass spectrometry (ICP-MS) and triple-quadrupole ICP-MS. The ^236^U activities and ^236^U/^238^U atom ratios in these soil samples were in the range of (0.469–24.4) × 10^−5^ Bq kg^−1^ and ((0.099–1.35) × 10^−7^), respectively. Higher ^240^Pu/^239^Pu atom ratios (0.245–0.312) and ^238^Pu/^239+240^Pu activity ratios (0.859–1.62) indicated Pu contamination originated from this accident and global fallout in some samples. For those soil samples along with black substances collected along roads in Fukushima Prefecture, high linear correlations were presented between ^236^U activities and ^239+240^Pu activities (Pearson’s r = 0.755, p < 0.01), and between ^236^U activities and ^238^Pu activities (Pearson’s r = 0.844, p < 0.01). The analysis of these soil samples confirmed the release of ^236^U, although in trace amounts, during the FDNPP accident.

## Introduction

The Fukushima Daiichi Nuclear Power Plant (FDNPP) accident in 2011 released huge amounts of radionuclides into the terrestrial environment. Related investigations have been focused on volatile fission and gamma-emitting radionuclides; however, the studies for nuclear fuel materials, such as Pu and U, have been limited^1–10^. For nuclear accidents, isotopic compositions and activity ratios of different radionuclides can provide important information on the situation of the reactor core, such as the fuel burn-up and the inventory of radionuclides in the reactor, and thus allow the investigation of the accident mechanisms^11^. ^236^U, ^239^Pu, and ^240^Pu observed in environmental and biological samples have been mainly introduced by human nuclear activities, such as nuclear weapon test explosions (global fallout), nuclear power generation and reprocessing of its fuel, and nuclear reactor accidents. In addition, ^236^U/^238^U and ^240^Pu/^239^Pu atom ratios vary with reactor, weapon, and fuel types^3,7,12–15^. Although only trace amounts of Pu and U were released from the FDNPP reactor cores^6–10^, this disaster has provided a rare opportunity to apply ^236^U/^238^U, ^240^Pu/^239^Pu, and ^236^U/^239^Pu atom ratios as tracers for contamination source identification in heavily contaminated samples. Heavy contamination mainly occurred northwest of the facility in a strip region approximately 40 km in length and 5 km in width, while most other areas of Fukushima Prefecture were only lightly contaminated^2^. The lightly contaminated regions have mixed contributions from both global fallout and the FDNPP accident fallout. Therefore, apart from studying the limited number of heavily contaminated samples to obtain the radionuclide signatures of this accident, it is also critical to study a larger number of other lightly contaminated samples from a wider region to establish a database for ^236^U/^238^U, ^240^Pu/^239^Pu, and ^236^U/^239^Pu for their risk assessment due to their long half-lives, and for their future application as tracers in environmental science.

For the measurement of ^236^U, inductively coupled plasma-mass spectrometry (ICP-MS), thermal ionization mass spectrometry (TIMS) and accelerator mass spectrometry (AMS) have usually been applied^16^. AMS is presently the method with the highest detection sensitivity for ^236^U measurement. However, due to the high equipment cost, there are only about 110 AMS facilities worldwide, and most of them are mainly applied to the routine analysis of ^14^C for dating purposes; only about ten of these instruments can be used to study ^236^U^9,10,12–25^. In addition, until now, three of four measurements regarding ^236^U for the FDNPP accident were performed at the same facility, the Vienna Environmental Research Accelerator (VERA) Laboratory at the University of Vienna, Austria^9,10,18^. Another measurement of ^236^U for the FDNPP accident was recently completed, using the low energy AMS system Tandy at ETH Zurich, Switzerland^26^. In addition, these analyses were mainly restricted to heavily contaminated road dust, aerosol samples, and litter with a smaller contribution by natural U in the matrix to avoid the dilution from natural U. In short, it is difficult to perform routine monitoring of ^236^U due to the FDNPP accident contamination using the available AMS facilities, even less establish the ^236^U/^238^U and ^236^U/^239^Pu database for future applications. However, from the viewpoint of risk assessment and protection against potential nuclear accidents, it is crucial to obtain sufficient ^236^U/^238^U and ^236^U/^239^Pu background data, since more nuclear power plants and nuclear waste processing facilities are being or will be built in the foreseeable future particularly in the northeastern Asian region.

Recently, Yang *et al*.^27^ have developed a novel method to measure trace ^236^U in environmental samples containing a larger natural U contribution. After total dissolution and chromatographic separation with one DGA resin column, they measured ^236^U/^238^U ratios as ^236^U^16^O^+^/^238^U^16^O^+^ by triple-quadrupole inductively coupled plasma-mass spectrometry (ICP-QQQ) analysis. The low detection limit (3.50 × 10^−6^ Bq kg^−1^ for ^236^U) of this method makes it possible to perform routine monitoring of environmental ^236^U originating from global fallout and FDNPP accident fallout.

Regarding the U released from the damaged reactor cores of the FDNPP, ^235^U and ^238^U have been measured in soil and plant samples collected from contaminated areas, but no anomaly of ^235^U associated with FDNPP fallout was observed because of the presence of a much larger quantity of natural U^28–30^. Although Sakaguchi *et al*.^18^ measured ^236^U/^238^Uatom ratios in seawater samples, an assessment of the ^236^U source was not possible. Recently, Schneider *et al*.^26^ studied vegetation, litter, and soil samples that had been collected in the vicinity of the damaged power plant in June 2013 and May 2015, however, even the highest ratio of (137 ± 6) × 10^−9^ for ^236^U/^238^U atom ratio in one soil sample could not be discriminated from the global fallout signature found in three surface soil samples in Japan ((6.18–10.9) × 10^−8^)^12,31^. In a word, the characteristics of U and transuranic elements as refractory elements released in the FDNPP accident have not been fully characterized in the environment, even six years after this accident. Therefore, more samples are required to identify the distribution of U and transuranic elements, and clarify their respective contributions from global fallout and FDNPP accident fallout.

The present study provides the results of actinide analyses conducted on 46 soil samples, with both heavy and light contamination by radiocesium due to the FDNPP accident, that were collected immediately after this accident. High resolution ICP-MS and ICP-QQQ were used to obtain the activities of ^239^Pu, ^240^Pu, and ^234^U, ^235^U, ^236^U, ^238^U, for the purpose of studying their distribution. Furthermore, the atom ratios of ^240^Pu/^239^Pu, ^236^U/^239^Pu, and ^236^U/^238^U were also calculated to quantify the potential contribution of the FDNPP accident. Finally, a preliminary database of ^236^U/^239^Pu and ^236^U/^238^U atom ratios in Fukushima Prefecture, Japan was built to investigate the relevance of using these as tracers of U and Pu in the future.

## Results

Activities were decay-corrected to the sampling date, activity or atom ratios were decay-corrected to March 11, 2011, to facilitate comparison with other studies. The ^134^Cs and ^137^Cs activities in these 46 soil samples were in the ranges from 12.5 to 1.10 × 10^5^ and from 14.1 to 1.10 × 10^5^ Bq kg^−1^-dry weight, respectively. The ^134^Cs/^137^Cs activity ratios were in a narrow range of 0.907–1.049 for 43 soil samples, and 0.047–0.489 for the other three soil samples with lower ^134^Cs activities of 13.8–81.1 Bq kg^−1^. The ^236^U activities and ^236^U/^238^U atom ratios in these 46 soil samples were in the range of (0.469–24.4) × 10^−5^ Bq kg^−1^ and ((0.099–1.35) × 10^−7^), respectively. In addition, the ^239+240^Pu activities and ^240^Pu/^239^Pu atom ratios in these soil samples ranged from 0.007 to 0.759 Bq kg^−1^-dry weight and from 0.162 to 0.312, respectively. ^238^Pu could only be determined in 5 samples, with activities of 0.055–0.470 Bq kg^−1^.

## Discussion

For all of the 46 soil samples collected in Fukushima Prefecture, significant ^134^Cs and ^137^Cs contamination from the FDNPP accident had been measured in the authors’ previous studies^4,5,32^. The ^134^Cs and ^137^Cs activities in these soil samples were in the ranges from 12.5 to 1.10 × 10^5^ and from 14.1 to 1.10 × 10^5^ Bq kg^−1^-dry weight, respectively. In a previous study, just following this accident, road dust particles, blackish in color, and commonly referred to as “black substances”, were collected and studied^10^. The black substances were composed of aerosol particles, asphalt particles, and minute tire particles originating from passing vehicles, as well as dried lichens, soil, and other fine-grained environmental debris. The dust, which was blown by wind and deposited by dry and wet precipitation into street corners and dips in the road, contained extremely high levels of radionuclides. The values in the present study were much lower than those found in the black substances which had ^134^Cs and ^137^Cs activities (decay-corrected to collection dates: from March to September, 2011) of (0.43–11.4) × 10^6^ and (0.58–17.7) × 10^6^ Bq kg^−1^, respectively^10^. Nevertheless, the ^134^Cs/^137^Cs activity ratios were in a narrow range of 0.907–1.049 for 43 soil samples, indicating obvious radiocesium contamination due to the FDNPP accident^33^. For the other three soil samples with lower ^134^Cs activities of 13.8–81.1 Bq kg^−1^, the ^134^Cs/^137^Cs activity ratios were found to be 0.047–0.489 due to the higher contribution from the global fallout instead of the FDNPP accident^5^. Since ^134^Cs (t_1/2_ = 2.06 y) in the environment before the FDNPP accident decayed out to undetectable level, the above data demonstrated that these samples indeed had been contaminated by radionuclides derived from this accident.

In contrast to radiocesium, mass spectrometric measurements of trace amounts of Pu and U isotopes in soil samples represent a greater challenge because of the large effects of polyatomic and isobaric interferences and the contribution of natural U and global fallout Pu and U isotopes. UO_2_ and mixed oxide ((U,Pu)O_2_) fuels had been used in the FDNPP reactors, with average ^235^U abundances from 3.4 to 3.7 wt% for the former and 1.2 wt% for the latter^34^. As shown in Table S1, the isotopic abundances of ^234^U, ^235^U, and ^238^U were calculated to be (1.96–10.8) × 10^−4^%, 1.69–1.90%, and 98.1–98.3% in the three damaged cores of Units 1, 2, and 3, respectively^34^. These values were significantly distinct from their natural isotopic abundances (0.005%, 0.720%, and 99.3% for ^234^U, ^235^U, and ^238^U, respectively). ^235^U/^238^U atom ratios ((7.17 ± 0.42) × 10^−3^) and ^234^U/^238^U atom ratios ((5.61 ± 0.46) × 10^−5^) in soil samples in the present study, along with data in soil samples contaminated by the FDNPP accident in previous studies^28–30^, were identical to their natural abundance ratios. Therefore, evidence of U release due to the FDNPP accident cannot be obtained by measuring ^234^U/^238^U and ^235^U/^238^U atom ratios, and if any occurred, the FDNPP-derived U was diluted largely by the higher amount of natural U in Japanese soil (about 1–3 ppm).

**Figure 1:**
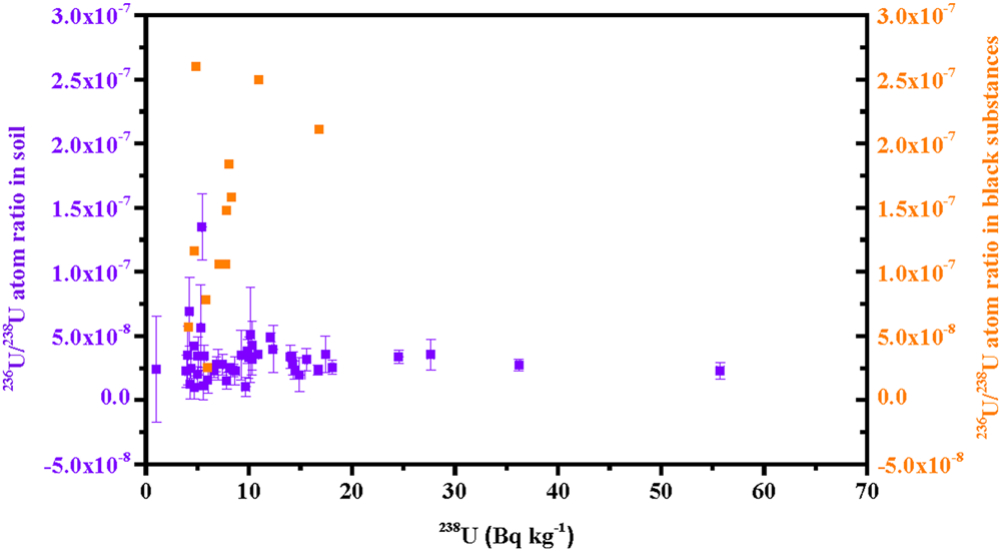
Plot showing the relationship between the ^238^U activities and the ^236^U/^238^U atom ratio in soil samples collected in Fukushima Prefecture immediately after the FDNPP accident (left ordinate) and in black substances collected along roads in Fukushima Prefecture (right ordinate)^10^. Error bars on the soil sample values correspond to 1σ.

As shown in Fig. 1, in these soil samples, the ^236^U/^238^U atom ratios ((0.099–1.35) × 10^−7^) were somewhat lower than that in the black substances ((0.25–2.60) × 10^−7^)^10^, and similar to those in litter and soil samples ((0.006–1.37) × 10^−7^)^26^. In addition, the ^236^U activities of (0.469–24.4) × 10^−5^ Bq kg^−1^ in soil could not be discriminated from those in black substances ((2.8–67.4) × 10^−5^ Bq kg^−1^)^10^ and in vegetable, litter, and soil samples (below the detection limit to 18.3 × 10^−5^ Bq kg^−1^)^26^. Regarding the U release from the FDNPP accident, the ^236^U/^238^U atom ratios could not be discriminated from global fallout signature found in three surface soil samples in Japan ((0.618–1.09) × 10^−7^)^12,31^ and in other areas^21,35,36^. In addition, there was no significant linear relationship between ^236^U activities and ^134^Cs activities in soil samples in the present study and in the black substances from a previous study^10^. Two reasons may explain this result: (1) the amount of released ^236^U from the FDNPP accident was significantly lower than that of ^134^Cs; (2) fractionation occurred between less volatile ^236^U and volatile ^134^Cs after release into the environment. Although the natural isotopic abundances of ^234^U and ^235^U are relatively lower than that of ^238^U, it was also impossible to indicate the release of ^236^U from the FDNPP accident via ^234^U/^236^U and ^235^U/^236^U atom ratios. All these indicated that the release of radioactive ^236^U from the FDNPP accident was in trace amounts for the studied areas even with heavy ^134^Cs contamination. In summary, it is impossible to confirm the release of ^236^U from the FDNPP accident only by comparing ^236^U with other uranium isotopes in the studied soil samples due to the mask effect of other uranium isotopes; however, the comparison of ^236^U with other radionuclides, such as ^239+240^Pu, may be plausible to confirm the ^236^U release, since these radionuclides have activities close in magnitude to those of ^236^U and have smaller contributions from natural sources.

Before the FDNPP accident, background information of Pu isotope activities and their atom ratios in Japanese soil samples were limited because of the challenge of Pu isotope analysis^37–41^. Recently, Yang *et al*.^3^ analyzed 80 surface soil samples collected from central-eastern Japan during 1969–1977 in order to establish the baseline before the FDNPP accident. In short, before this accident, ^239+240^Pu activities in the surface soil samples from agricultural fields, forests, school grounds, parks, and residential areas were found to be in the range of 0.004−4.31 Bq kg^−1^-dry weight^3,37–41^. After this accident, ^239+240^Pu activities in the surface soil samples collected in Fukushima Prefecture were still quite low, ranging from 0.007 to 0.759 Bq kg^−1^-dry weight, without significant change compared to the background values (0.050–0.695 Bq kg^−1^-dry weight) before this accident^3^. Among the 46 soil samples analyzed here, the ^239+240^Pu activities for 28 of these soil samples were lower than 0.100 Bq kg^−1^-dry weight. Therefore, from the viewpoint of ^239+240^Pu activities, it is impossible to conclude firmly from this soil sample study that some fractions of Pu were released into the environment during the FDNPP accident.

**Figure 2:**
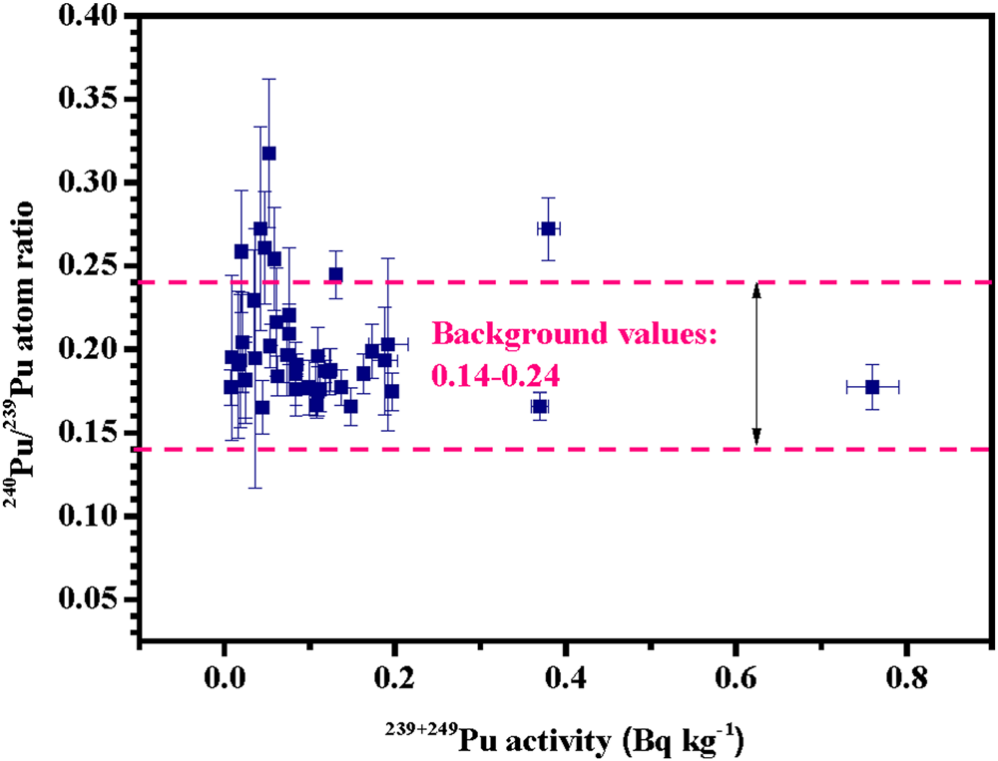
Plot showing the relationship between the ^240^Pu/^239^Pu atom ratio and the ^239+240^Pu activity in soil samples collected in Fukushima Prefecture immediately after the FDNPP accident. Error bars on the soil sample values correspond to 1σ.

**Figure 3:**
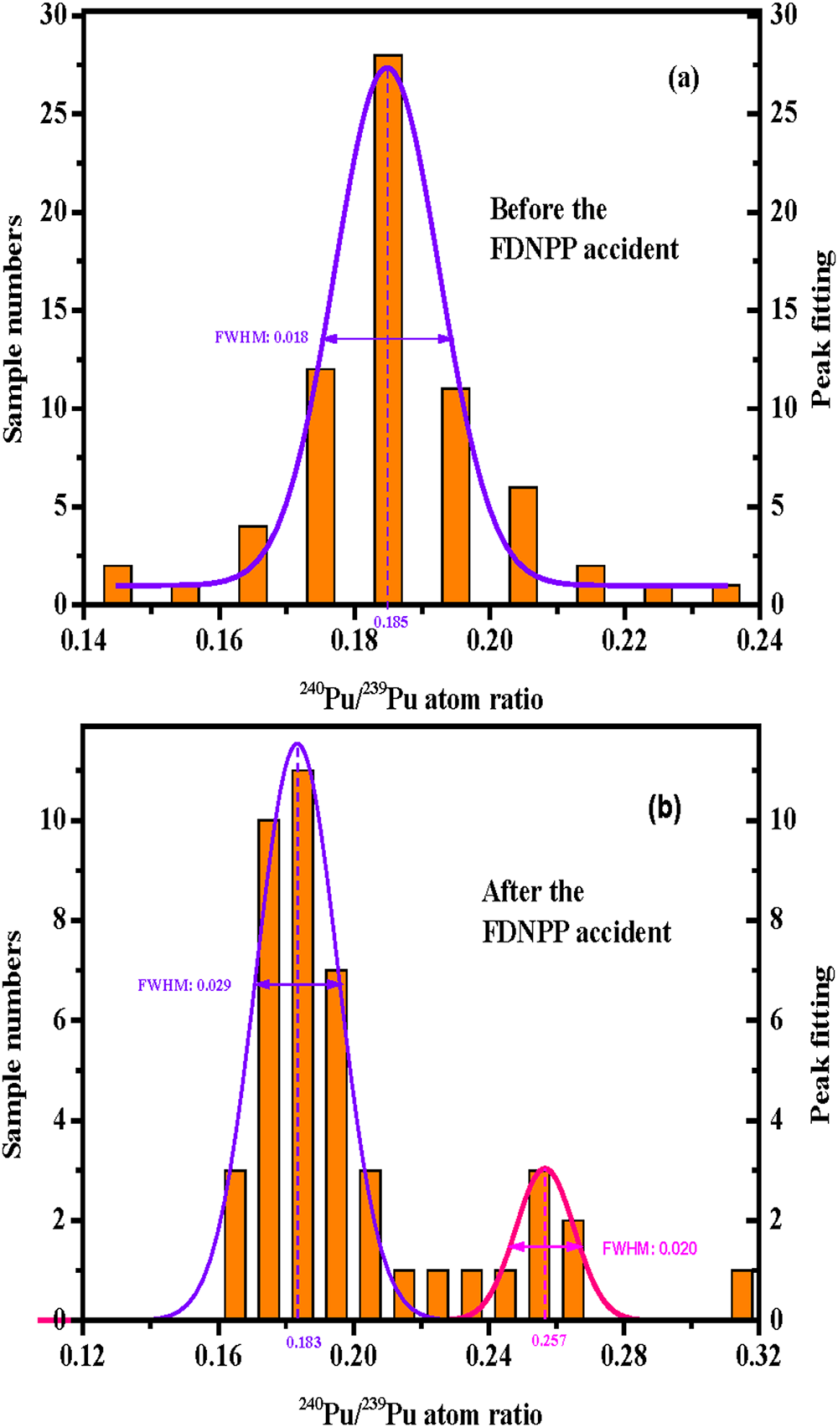
Frequency distributions of ^240^Pu/^239^Pu atom ratios (**a**) in soil samples before the FDNPP accident^3^ and (**b**) in soil samples after this accident (FWHM: full width at half maximum).

Before the FDNPP accident, the ^240^Pu/^239^Pu atom ratios in the soil samples were found to be in the range of 0.14–0.24, indicating that the major source was global fallout due to atmospheric nuclear test explosions conducted in the last century^3,37–42^. In the present study, the observed ^240^Pu/^239^Pu atom ratios were in a wider range, 0.162–0.312 as shown in Fig. 2. Nishihara *et al*.^34^ have estimated the possible ^240^Pu/^239^Pu atom ratios that existed in March 2011 in the FDNPP Units 1 to 3 reactor cores (0.320–0.356) and their spent fuel pools (0.394–0.468) using the ORIGEN2 code and the fuel burn-up data from the Tokyo Electric Power Company (Table S1). Furthermore, Zheng *et al*.^8^ estimated that a trace amount of Pu isotopes (~2 × 10^−5^% of core inventory) was released into the environment only from the damaged reactors, but not from the spent fuel pools. Though limited in number, one soil^7^, four litter^7,43^, seven black substance^43^, and three aerosol^9^ samples were reported as having ^240^Pu/^239^Pu atom ratios in a narrow range (0.286–0.365), and that were similar to the expected value in the reactor cores. One aerosol sample (0.426 ± 0.057)^9^, two vegetation samples (0.381 ± 0.046 and 0.64 ± 0.37)^42^, and recent study in vegetable, litter, and soil samples^26^ presented higher ^240^Pu/^239^Pu atom ratios but with larger errors. Accordingly, these data were not considered in the following discussion. Finally, the ^240^Pu/^239^Pu atom ratios with small uncertainties of 0.286–0.365 were considered to represent the Pu isotope signature regarding of the FDNPP accident fallout. In the present study, 7 soil samples (about 15% of the samples) showed higher ^240^Pu/^239^Pu atom ratios than the background values (0.14–0.24), as shown in Fig. 2 and Table S2. In the authors’ previous study, the frequency distribution of ^240^Pu/^239^Pu atom ratios was shown as a unimodal pattern (Fig. 3a) (median, 0.185, FWHM, 0.018) before this accident^3^. Because of the additional Pu release from the FDNPP accident, the frequency distribution of ^240^Pu/^239^Pu atom ratios became a bimodal one after this accident (Fig. 3b) (median, 0.183, HWHM, 0.029; and median, 0.257, HWHM, 0.020). After the FDNPP accident as shown in Fig. 3b, the first peak value for the ^240^Pu/^239^Pu atom ratios (0.162–0.237) was close to that value before it. The ^240^Pu/^239^Pu atom ratios of the maximum peaks for these two curves were 0.185 and 0.183, respectively. All these findings indicated the first peak was due to global fallout with insignificant Pu contamination from the FDNPP accident. The second peak (0.245–0.312) was located between the global fallout and the FDNPP accident signature values, indicating mixed Pu contributions from both sources.

The activity ratio of ^238^Pu/^239+240^Pu is also important and has been demonstrated to be useful to distinguish many prominent Pu sources in the environment^8^. Among 46 soil samples, ^238^Pu could only be determined in 5 samples (0.055–0.470 Bq kg^−1^) (Table S2). The activity ratios of ^238^Pu/^239+240^Pu in these 5 samples ranged from 0.859 to 1.62, which were much higher than values for the global fallout (0.032), the atmospheric fallout from the year 1963–1979 in Japan (0.037), the Nagasaki A-bomb (0.074 ± 0.001), the Pacific Proving Ground tests (0.001–0.014), and the Chernobyl accident (0.5)^8^. In addition, the activity ratios of ^238^Pu/^239+240^Pu in the present study remained below the corresponding values in the fuels in the reactor cores of Units 1 to 3 (2.31–2.92)^34^. Therefore, the activity ratios of ^238^Pu/^239+240^Pu also indicated clearly a mixed contribution from both global fallout and the FDNPP accident fallout in the studied samples.

**Figure 4:**
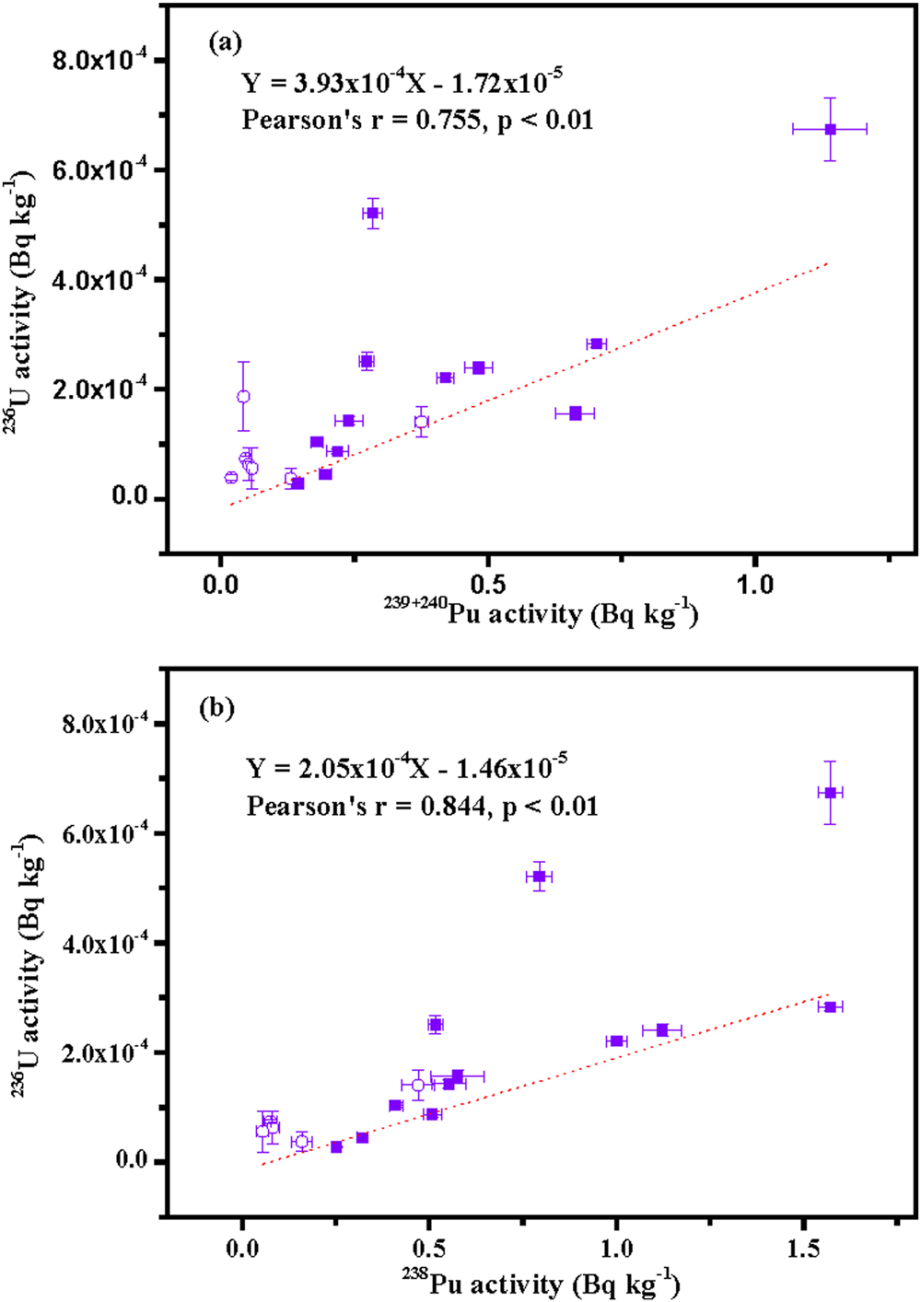
For the soil samples with a clear Pu contamination due to the FDNPP accident (^240^Pu/^239^Pu atom ratios, 0.245–0.312, and ^238^Pu/^239+240^Pu activity ratios, 0.859–1.62) in the present study (circle symbols), as well as black substances (square symbols) collected along roads in Fukushima Prefecture^10^, strong linear correlations were derived for: (**a**) ^236^U activities and ^239+240^Pu activities; and (**b**) ^236^U activities and ^238^Pu activities. Error bars on the sample values correspond to 1σ.

**Figure 5:**
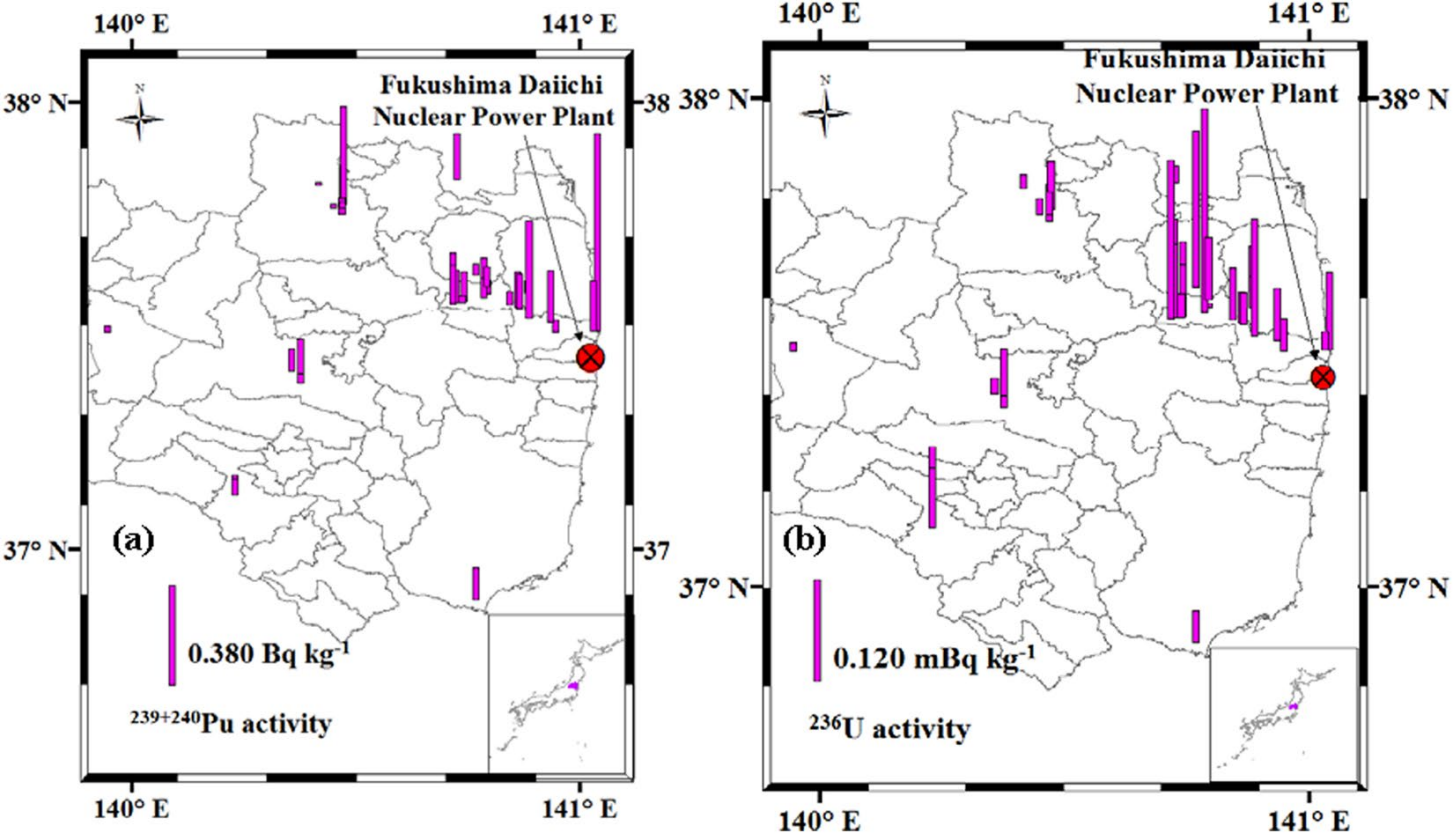
^236^U and ^239+240^Pu activity distributions in soil samples collected in Fukushima Prefecture immediately after the FDNPP accident. This map was prepared with Arc GIS 10.3 software.

The ^236^U/^239^Pu atom ratios in the Fukushima soil samples varied between 0.147 and 8.44. On the other hand, the ^236^U/^239^Pu atom ratio values in global fallout in the Northern Hemisphere have been generally documented to be in the range of 0.04–0.78^12,21,35,36^. From the frequency distribution of ^236^U/^239^Pu atom ratios shown in Figure S1, only 10 samples analyzed in the current research were characterized by values in the global fallout range. Since ^236^U data are limited in environmental samples, the data of black substances reported by Sakaguchi *et al*.^10^ were also included in the following discussion to describe the linear relationship between ^236^U and Pu isotopes, as shown in Fig. 4. For the soil samples with obvious Pu contamination from the FDNPP accident (^240^Pu/^239^Pu atom ratios, 0.245–0.312, and ^238^Pu/^239+240^Pu activity ratios, 0.859–1.62), a moderate linear correlation was presented between ^236^U activities and ^239+240^Pu activities (Pearson’s r = 0.567) (Table S2), and the linear correlation became stronger when data from black substances were added (Pearson’s r = 0.755, p < 0.01) (Fig. 4a). For these soil samples, a moderate linear correlation was also presented between ^236^U activities and ^238^Pu activities (Pearson’s r = 0.567) (Table S2), and the linear correlation became stronger when data from black substances were added (Pearson’s r = 0.844, p < 0.01) (Fig. 4b). These clearly indicated that ^236^U was indeed released during the FDNPP accident with Pu isotopes and this was confirmed through the analysis of the soil samples. These also indicated somewhat light fractionation between Pu and U in the studied soil samples. It should be noted that both U and Pu were affected by global fallout and the FDNPP accident fallout. Although nearly identical ^236^U/^239+240^Pu activity ratios have been found in the 0–30 cm depth soil layer ((1.18 ± 0.04) × 10^−4^)^31^, spatial distributions of ^236^U and ^239+240^Pu activities, as shown in Fig. 5, revealed that a different distribution between ^239+240^Pu and ^236^U was found in the soil samples studied. The Pu contamination of surface soil samples decreased rapidly with the distance from the FDNPP (Fig. 5a), while the ^236^U contamination of the surface soil samples increased first in the northwest direction from this facility (Fig. 5b). In summary, the analysis of these soil samples confirmed the release of ^236^U, although in trace amounts, during the FDNPP accident. In the future, analyses by new techniques of more samples collected in a wider region are highly required to show the distinct distribution of ^236^U and ^239+240^Pu in Japan comprehensively.

## Materials and Methods

### Soil Sampling

The procedure details for soil sampling and pre-treatment have been described elsewhere^32^, and the sample locations can be found in Table S4. Surface soils (0–5 cm) were collected from 46 sites in Fukushima Prefecture (Fig. 5) during five sampling expeditions conducted in 2011, from March 17 to 19, April 12 to 16, April 26 to 27, June 6 to 16, and July 18 to 19, respectively. The collection sites were mainly restricted to the heavily contaminated region where the radioactive plume due to the FDNPP accident was washed out by rainfall. Fukushima Prefecture is divided by mountain ranges into three regions (from west to east) showing large temperature and weather contrasts^44^. On average, annually, central Fukushima receives 1166 mm of precipitation and 189 cm of snow, respectively.

After large particles and plant roots were removed by handpicking, soil samples were transferred into 100-mL polystyrene containers, and then, only the fine fraction of soil particles (diameter below 2 mm) was analyzed.

### Analysis of U Isotopes

The separation and purification procedure for U isotopes was conducted following the method of Yang *et al*.^27^. About 1 g soil samples were ashed in a muffle oven at 450 °C for 2 h to decompose organic matter. Total dissolution (HF + HNO_3_ + HClO_4_) was performed in PFA jars with lids (Savillex, Eden Prairie, MN, USA) on a hot plate at 180 °C for 1 d. After filtration, Si was removed by reaction with 46% HF. Then, the HF solution was heated to dryness, after which 5 mL of 61% HNO_3_ was added and this acid solution was heated to dryness to remove residual HF. Subsequently, the sample residue was dissolved into 10 mL of 6 M HNO_3_, ready for chromatographic purification using DGA resin. After removing interfering elements by 6 M HNO_3_ and 8 M HNO_3_, U was eluted from the resin by 15 mL of 0.1 M HNO_3_. Finally, the U eluate was evaporated to near dryness and dissolved into 1.5 mL of 4% HNO_3_. A 20 μL aliquot was taken out and diluted with 4% HNO_3_ at a dilution factor of 2000 for the ^238^U concentration (activity) measurement via an Agilent 8800 ICP-QQQ operated in the single MS mode (Agilent Technologies, Santa Clara, CA, USA). The remaining portion was analyzed for ^234^U/^238^U, ^235^U/^238^U, and ^236^U/^238^U atom ratios, via the Agilent 8800 ICP-QQQ MS/MS mode operation. Finally, ^234^U, ^235^U, ^236^U activities could be calculated by combining the data of these two mode analyses.

### Analysis of Pu Isotopes

Preparation and purification of Pu isotopes were conducted based on previous study with a modification from the method of MEXT (Ministry of Education, Culture, Sports, Science and Technology, Japan)^7^. Briefly, 10 g samples were ashed in a muffle oven at 500 °C for 3 h to decompose organic matter. The ashed samples were digested by heating on a hot plate using 10 M HNO_3_-1M HF. After filtration, the Pu solution was loaded on the column and purified by anion-exchange chromatography (Dowex 1 × 8) and then electrodeposited onto a stainless steel disc. The activities of ^238^Pu and ^239+240^Pu were measured with α-spectrometer. Then, Pu on the stainless-steel disc was re-dissolved with 10 M HNO_3_‐1 M HF. Pu isotopes in this solution were further purified by anion-exchange chromatography. After loading the sample solution on the first Dowex 1 × 8 column (6 mL), sequential elution of U, Th and Pu was conducted using 120 mL of 8 M HNO_3_, 150 mL of 10 M HCl, and 100 mL NH_4_I-HCl solution, respectively. After adding 5 mL HNO_3_ to the final eluate, the obtained Pu fraction was heated to dryness, and the residue was dissolved into 10 mL of 4 M acetic acid. This solution was then loaded onto the second Dowex 1 × 8 column (2 mL) and 20 mL of 4 M acetic acid was used to rinse the column. The effluents of both loading and rinse solutions were collected (30 mL) for Pu analysis. The collected acetic acid solution was heated to dryness and the residue was dissolved into 10 mL of 4% HNO_3_ for ^239^Pu and ^240^Pu analysis via an APEX-Q/SF-ICP-MS (ELEMENT 2, Thermo Fisher Scientific, Bremen, Germany). The chemical yields of Pu were determined by using a ^242^Pu yield tracer with negligible quantities of ^239^Pu and ^240^Pu. The data of Pu isotopes were obtained after blank correction. As shown in Table S3, during analysis, several samples were randomly selected for α-spectrometry measurement and compared with the results of ICP-MS for method validation.

## Electronic supplementary material

Supplementary Information
