# Supplementary material for: Isotopic compositions of 236U, 239Pu, and 240Pu in soil contaminated by the Fukushima Daiichi Nuclear Power Plant accident
Source: Sci Rep. 2017 Oct 19;7:13619. doi: 10.1038/s41598-017-13998-6 (PMC5648813; doi:10.1038/s41598-017-13998-6)
Supplement: Supplementary file 1 — Supplementary Information [file 41598_2017_13998_MOESM1_ESM.pdf]

Supporting Information

Isotopic compositions of ^{236}U, ^{239}Pu, and ^{240}Pu in soil contaminated by the Fukushima Daiichi Nuclear Power Plant accident

Guosheng Yang [1,2], Hirofumi Tazoe[1], Kazuhiko Hayano[3], Kumiko Okayama[3], Masatoshi Yamada[1,*]

[1]Department of Radiation Chemistry, Institute of Radiation Emergency Medicine, Hirosaki University, 66-1 Hon-cho, Hirosaki, Aomori 036-8564, Japan

[2]Division of Nuclear Technology and Applications, Institute of High Energy Physics, Chinese Academy of Sciences; Beijing Engineering Research Center of Radiographic Techniques and Equipment, Beijing 100049, China

[3]Mutsu Analytical Sciences Laboratory, Japan Chemical Analysis Center, 4-24 Minatomachi, Mutsu, Aomori 035-0064, Japan

Number of pages	Number of tables	Number of figures
8	4	1

*Corresponding author. Tel.: +81 172 39 5405; Fax: +81 172 39 5405.

myamada@hirosaki-u.ac.jp

Table S1. Pu and U isotopic compositions in fuels loaded in three reactor cores of the Fukushima Daiichi Nuclear Power Plant, cited from the literature [1]. The reference gave no uncertainties for the values.

	**Core 1**	**Core 2**	**Core 3**
	**Isotopic abundance (%)**		
^{234}U	3.19×10^{-4}	1.96×10^{-4}	1.08×10^{-3}
^{235}U	1.69	1.90	1.88
^{238}U	98.3	98.1	98.1
	**Atom ratio**		
$^{240}Pu/^{239}Pu$	0.343	0.319	0.355
$^{236}U/^{239}Pu$	0.804	0.789	0.639
	**Activity ratio**		
$^{236}U/^{239+240}Pu$	3.67×10^{-4}	3.75×10^{-4}	2.86×10^{-4}
$^{238}Pu/^{239+240}Pu$	2.92	2.39	2.31

Table S2. The details of U and Pu isotopes for the soil samples with clear Pu contamination due to the FDNPP accident ($^{240}Pu/^{239}Pu$ atom ratio, 0.245−0.312; $^{238}Pu/^{239+240}Pu$ activity ratios, 0.859−1.62). Error bars on the soil sample values indicate 1σ.

**Sample**	**$^{240}Pu/^{239}Pu$ atom ratio**	**^{238}Pu activity (Bq kg^{-1})**	**$^{239+240}Pu$ activity (Bq kg^{-1})**	**^{236}U activity (×10^{-5} Bq kg^{-1})**	**$^{236}U/^{238}U$ atom ratio (×10^{-8})**
S12	0.256±0.034	0.076±0.018	0.047±0.003	7.36±0.35	3.55±0.16
S46	0.258±0.037		0.020±0.001	3.91±0.50	2.53±0.32
S54	0.268±0.060		0.043±0.005	18.7±6.3	3.53±1.19
S273	0.245±0.014	0.160±0.027	0.131±0.004	3.74±1.82	2.27±1.09
S275	0.312±0.044	0.081±0.019	0.052±0.004	6.32±3.05	3.32±1.60
S277	0.255±0.030	0.055±0.016	0.059±0.003	5.59±3.78	1.97±1.33
S279	0.267±0.018	0.470±0.045	0.375±0.013	14.1±2.7	13.5±2.6

Table S3. The results of α-spectrometry and ICP-MS for ^{239}Pu and ^{240}Pu. Error bars on the soil sample values indicate 1σ.

**Method**	**ICP-MS**		**α-spectrometry**
**Sample**	**^{239}Pu activity (Bq kg^{-1})**	**^{240}Pu activity (Bq kg^{-1})**	**$^{239+240}Pu$ activity (Bq kg^{-1})**
S12	0.024±0.002	0.023±0.002	0.047±0.003
S273	0.069±0.001	0.062±0.003	0.120±0.022
S275	0.024±0.001	0.028±0.004	0.059±0.015
S277	0.030±0.001	0.028±0.003	0.064±0.015
S279	0.189±0.011	0.186±0.007	0.430±0.043

Table S4. Collection locations of soil samples.

ID	Latitude (°N)	Longitude (°E)	Collection date (year/month/day, time)
S2	37.3944	140.3752	2011/3/17 15:27
S3	37.7628	140.4685	2011/3/17 17:40
S6	36.8886	140.7674	2011/3/18 14:30
S7	37.4847	139.9451	2011/3/18 17:35
S8	37.1221	140.2285	2011/3/19 13:15
S9	37.8278	140.7239	2011/3/19 15:45
S12	37.5744	140.8822	2011/4/12 16:15
S13	37.5744	140.8822	2011/4/12 16:20
S14	37.5419	140.8594	2011/4/12 16:51
S15	37.5733	140.7931	2011/4/13 11:20
S16	37.6036	140.7844	2011/4/13 13:00
S19	37.5538	140.7216	2011/4/13 21:50
S20	37.5538	140.7216	2011/4/13 21:56
S23	37.5538	140.7216	2011/4/13 22:10
S27	37.5538	140.7216	2011/4/13 22:44
S29	37.5538	140.7216	2011/4/13 23:10
S35	37.5538	140.7216	2011/4/14 00:06
S36	37.5538	140.7216	2011/4/14 00:30
S37	37.5538	140.7216	2011/4/14 00:50
S39	37.5561	140.7398	2011/4/14 03:00
S40	37.5519	140.7159	2011/4/14 20:30
S42	37.5519	140.7159	2011/4/14 21:00
S44	37.5519	140.7159	2011/4/14 21:20
S45	37.5561	140.7398	2011/4/15 00:30
S46	37.5651	140.7839	2011/4/15 03:46
S48	37.5651	140.7839	2011/4/15 06:20
S49	37.5881	140.7919	2011/4/15 12:20
S54	37.6161	140.7672	2011/4/16 03:04
S55	37.1221	140.2285	2011/4/26 16:50
S57	37.3706	140.3752	2011/4/27 01:16
S58	37.7628	140.4685	2011/4/27 06:30

S60	37.4847	140.9451	2011/4/27 21:00
S63	37.7741	140.4710	2011/6/6 17:30
S65	37.7508	140.4679	2011/6/6 18:20
S66	37.7637	140.4686	2011/6/7 17:20
S81	37.7643	140.4483	2011/6/9 13:30
S83	37.8166	140.4149	2011/6/9 13:55
S92	37.3980	140.3560	2011/6/16 15:30
S273	37.5402	140.8646	2012/7/18 10:00
S275	37.5484	140.8422	2012/7/18 11:15
S276	37.5538	140.7365	2012/7/18 13:20
S277	37.5338	140.8685	2012/7/19 10:50
S279	37.5165	140.8854	2012/7/19 11:10
S280	37.5076	140.9330	2012/7/19 11:40
S282	37.4876	141.0383	2012/7/19 13:15
S283	37.4872	141.0297	2012/7/19 14:40

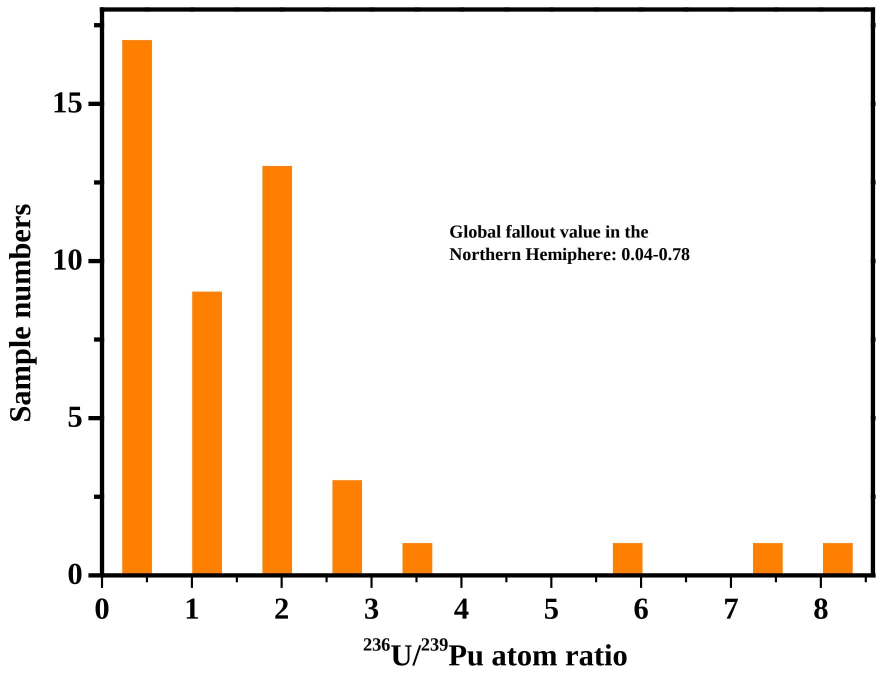

Figure S1. The frequency distribution of $^{236}U/^{239}Pu$ in soils samples collected after the FDNPP accident.

References

1. Nishihara, K., Iwamoto, H. & Suyama, K. Estimation of fuel compositions in Fukushima-Daiichi Nuclear Power Plant. JAEA-Data/Code 2012-018 (2012).
